# Translation, cultural adaptation, and evidence of content and face validity of a questionnaire to assess REDs knowledge in Brazil: a cross-cultural validation study

**DOI:** 10.1590/1516-3180.2025.3135.08042026

**Published:** 2026-07-17

**Authors:** Uyara Pereira de Maria, Claudia Ridel Juzwiak

**Affiliations:** I Doctoral Student, Interdisciplinary Postgraduate Program in Health Sciences, Universidade Federal de São Paulo (Unifesp), Santos (SP), Brazil.; II Professor, Department of Human Movement Sciences, Universidade Federal de São Paulo (Unifesp), Santos (SP), Brazil.

**Keywords:** Validation study, Athletes, Female athlete triad syndrome, Validation studies, Translating, Cross-cultural adaptation, Athlete, Relative energy deficiency in sport, Knowledge

## Abstract

**BACKGROUND:**

Relative energy deficiency in sport (REDs) is a syndrome that impairs physiological function because of low energy availability. Awareness and knowledge of REDs among athletes and health professionals are essential for early identification and prevention.

**OBJECTIVE:**

To translate into Brazilian Portuguese and culturally adapt a questionnaire that assesses knowledge of the signs, symptoms, and consequences of REDs, originally created by Pai et al., and to obtain evidence of content and face validity.

**DESIGN AND SETTING:**

Translation, cultural adaptation, and validation study conducted remotely using online tools and digital data collection.

**METHODS:**

The translation and cultural adaptation consisted of the following steps: two independent translations, synthesis of the translations by a committee of health professionals, two back-translations, and review by a committee of experts to determine the best version for testing. For content validity, the content validity coefficient (CVC) was calculated based on the evaluation of the experts committee on the practical pertinence, theoretical relevance, and linguistic clarity of the items of the pretest version. For face validity, the pretest version was applied to 30 individuals who assessed the items for linguistic clarity, adequacy, and comprehensibility.

**RESULTS:**

The CVC for the scale was 0.97. The CVC values for clarity, pertinence, and relevance were 0.97, 1 and 0.99, respectively. All items had a CVC > 0.8, confirming acceptable content validity. The pretest version was tested on 12 female runners, eight coaches, and 10 dietitians. Items were clear and comprehensible to 93% and adequate to 96% of respondents. No changes were needed, and the pretest version was defined as the Brazilian version of the questionnaire to assess REDs knowledge (QC-REDs).

**CONCLUSION:**

The QC-REDs obtained strong content and face validity. It provides a rigorously adapted tool for Brazil, supported by positive feedback from the target population, demonstrating its applicability and usability. This instrument fills a crucial gap and offers a valuable resource for researchers and professionals working with athletes at risk of REDs. Future research should focus on psychometric validation to further solidify its effectiveness in the Brazilian context.

## INTRODUCTION

 Relative Energy Deficiency in Sport (REDs) was first described in 2014 in a position statement by the International Olympic Committee (IOC) as a syndrome characterized by low energy availability (LEA) as its central component.^1^

 LEA refers to insufficient energy availability to support physiological functions after considering energy expended during exercise,^2^ and has been suggested to occur in women when it is equal to or less than 30 kcal/kg FFM/day and in men when equal to or less than 9–25 kcal/kg FFM/day.^3-7^ LEA’s effects can vary based on the severity, duration, and frequency of exposure, with adaptable LEA causing minimal or no impact, whereas problematic LEA leads to persistent disruptions across various body systems.^8^ Therefore, the more severe and prolonged the LEA, the greater the risk of leading to a persistent disruption of hormonal, metabolic, and physiological regulations,^9-11^ acting as an etiological factor for REDs. 

 REDs has deleterious effects on multiple physiological functions, including menstrual dysfunction, impaired reproductive function, and impairments in several systems, such as bone health, cardiovascular and neurocognitive function, energy metabolism, immunity, and skeletal muscle function. Sports performance is also reduced, manifested as decreased endurance and power, reduced muscle strength, impaired recovery, motivation, and training response, in addition to decreased cognitive performance.^8^

 The identification and management of REDs are critical aspects of athlete health; however, research shows a concerning knowledge gap among sports coaches and other health professionals, such as physiotherapists and physicians.^12^ A study found that fewer than 30% of health professionals, including certified athletic trainers, were familiar with the term REDs after attending a sports medicine conference.^12^ In addition, even among physicians trained in sports medicine and other health professionals, few expressed confidence in treating patients with REDs.^12^ This lack of awareness is particularly troubling, as these professionals are uniquely positioned within the technical team to identify athletes at risk and educate them on the importance of maintaining adequate energy availability.^8^ Moreover, athletes often have limited knowledge about REDs, with many unaware of the connection between inadequate nutrition and menstrual dysfunction, for example, which can lead to serious long-term health consequences.^13^

 In the absence of a validated questionnaire designed to evaluate the level of knowledge about REDs among stakeholders in the sports environment, Pai et al.^14^ developed and validated a questionnaire with satisfactory validity evidence to assess knowledge of the signs and symptoms of REDs. The instrument includes items that identify awareness of the female athlete triad, LEA, and REDs, as well as the recognition of REDs-related symptoms and their health and performance consequences. When applying this questionnaire, Pai et al.^14^ observed that health professionals exhibited higher levels of knowledge about REDs than physically active individuals. This finding suggests that the instrument can be a valuable tool for assessing current knowledge on REDs and identifying gaps that need to be addressed.^14^

 To the authors’ knowledge, no validated questionnaire is available to assess knowledge of the signs, symptoms, and consequences of REDs among Brazilian health professionals and athletes. Given the severity of the consequences of LEA-related consequences, particularly in the development of REDs, raising awareness about this syndrome should be the first step toward prevention. Therefore, adapting and validating a questionnaire to assess knowledge of REDs within the Brazilian sports stakeholder community is crucial for advancing awareness and improving strategies for disseminating information about REDs in both sports and academic settings. In this context, this study aims to translate the questionnaire designed by Pai et al.^14^ into Brazilian Portuguese, culturally adapt it, and obtain content and face validity evidence for its use in Brazil. 

## MATERIAL AND METHODS

 This cross-sectional study for linguistic and cross-cultural validation was reviewed and approved by the Ethics and Research Committee of the Universidade Federal de São Paulo (No. 67851223.1.0000. 5505), and all participants provided informed consent. 

 The questionnaire to assess REDs knowledge is a self-administered instrument that was first developed in English and consists of 18 items assessing the theoretical and practical understanding of REDs’ signs, symptoms, and consequences.^14^ The original questionnaire was validated in a sample (n = 174) composed of health professionals and physically active individuals from New Zealand, aged between 18 and 70 years.^14^ The instrument developed by Pai et al.^14^ has a dichotomous format, with results classified as correct (score = 1) or incorrect (score = 0). Thus, comprising 18 scoring items, the instrument’s total score ranges from 0 to 18. Higher scores indicate a higher level of knowledge about REDs. 

**Figure 1:**
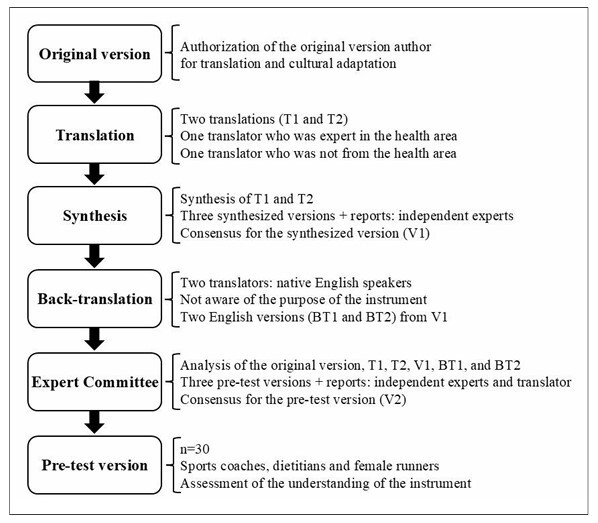
Translation and adaptation process, followed by evidence of validity.

 To initiate the translation and adaptation process, permission to use the questionnaire to assess REDs knowledge was first obtained by email from the corresponding author of the original version. The process of translating, culturally adapting, and validating the content of the questionnaire was carried out based on the recommendations of Guillemin et al.^15^ and Beaton et al.^16^ (**Figure 1**). 

 The original version of the questionnaire was translated into Portuguese by two independent Brazilian translators fluent in English. Translator 1 was a registered dietitian with a master’s degree in the LEA area and an expert on instrument construct. Translator 2 was an accredited translator with command of both English and Portuguese and no knowledge of the questionnaire’s construct. The two versions in Portuguese (T1 and T2) were independently analyzed by three registered sports dietitians knowledgeable about REDs (two with a master’s degree and one PhD professor) to determine the best wording for the target population. The experts assessed the equivalence between the translations and the original instrument across four aspects: (1) semantic equivalence, to determine whether the wording had the same meaning and to identify any grammatical errors; (2) idiomatic equivalence, to evaluate whether expressions that were difficult to translate were well adapted without changing the cultural meaning of the item; (3) experiential equivalence, to assess the applicability of the items in the new cultural context and, if necessary, replace them with equivalent items; and (4) conceptual equivalence, to ensure that terms and expressions, even if accurately translated, measured the same construct across different cultures.^17^ The three versions were synthesized by the authors into a single Portuguese version (V1). 

 Subsequently, version V1 was subjected to back-translation (translation from Portuguese to English) by two native Englishspeaking translators with command in Portuguese and no prior knowledge of the instrument’s construct, resulting in two English versions, BT1 and BT2. Next, a committee of experts – comprising four health professionals and/or researchers from the health area with knowledge of the construct and fluency in English, as well as one of the translators involved in the process – independently evaluated all the materials produced during the process (original, T1, T2, V1, BT1, and BT2) to develop the pretest version. The committee included three sports registered dietitians and one sports gynecologist. Each member created a pretest version and provided a report on the process. These versions and reports were then compared by the authors, and based on theoretical experts’ consensus, the final pretest version (V2) was structured. 

 The content validity process consisted of the evaluation of the materials generated from the translation, synthesis, and backtranslation stages by an expert committee. The experts assessed the items of the synthesis version (V1) based on practical pertinence, theoretical relevance, and linguistic clarity, ensuring that the instrument effectively measured its intended construct.^18^ Each item was rated using a five-point Likert scale for each criterion (clarity, pertinence, and relevance), where “1” indicated that the item was not at all clear, pertinent, or relevant, whereas “5” indicated that the item was completely clear, pertinent, or relevant. These ratings enabled the calculation of the content validity coefficient (CVC).^19^

 To calculate the CVC of the instrument, as well as the CVC for each item and each evaluated aspect related to the scale construct, the equations proposed by Filgueiras et al.^20^ were applied. According to Hernández-Nieto,^19^ instrument items should have a minimum CVC value of 0.8. However, values above 0.7 are considered acceptable.^19^

 Furthermore, face validity concerns whether the target population considers the items understandable, relevant, and easy to answer.^21^ Therefore, face validity was assessed by testing the pretest version with female runners, running coaches, and registered dietitians using a non-probabilistic snowball sampling method. Participants were invited via social media and the researchers’ professional networks. As recommended by Beaton et al.,^16^ 30 individuals participated in the face validity assessment. While completing the questionnaire, participants were asked to evaluate the clarity, adequacy, and comprehensibility of each item and to suggest modifications when necessary to improve understanding. Items that elicited doubts or were not understood by more than 15% of respondents were flagged for revision, and any modifications required would necessitate reapplication of the updated version to new respondents.^22^ The questionnaire was applied online, and data were collected and managed using the Research Electronic Data Capture (REDCap) platform hosted at Universidade Federal de São Paulo.^23,24^

## RESULTS

 The Content Validity Coefficient (CVC) for the entire scale, calculated by subtracting the mean experts’ CVC for the scale as a whole from the experts’ standard error of polarization, was 0.97. For the clarity of the items, the CVC was 0.97. For the pertinence of the items, that is, their importance in composing the instrument, the CVC value was 1. Finally, for the relevance of the items in measuring the construct, the CVC was 0.99. **Table 1** presents the pretest version and the CVC value of each item according to each aspect judged by the five experts. However, the translator on the expert committee preferred to evaluate only the clarity of the items, considering himself unqualified to assess items within the construct that were not part of his area of expertise. Therefore, the CVC values for pertinence and relevance were calculated based on the judgments of four experts. All items had a CVC > 0.8, indicating that the pretest version of the instrument demonstrated acceptable content validity. 

**Table 1:** Calculation of CVC of the pretest version items

**Pretest version (V2)**		**CVC**		
		**Clarity**	**Pertinence**	**Relevance**
Você já ouviu falar de Baixa Disponibilidade de Energia?				
	• Sim	0,96	1	1
	• Não			
Você já ouviu falar de Deficiência Relativa de Energia nos Esportes?				
	• Sim	1	1	1
	• Não			
Você já ouviu falar de Tríade da Mulher Atleta?				
	• Sim	1	1	1
	• Não			
Ter um ciclo menstrual irregular costuma ser um sinal de que mulheres atletas/fisicamente ativas estão no auge da forma competitiva				
	• Verdadeiro	0,96	1	1
	• Falso			
	• Não tenho certeza			
Você acha normal mulheres atletas/ fisicamente ativas não menstruarem? (excluindo gravidez ou ausência de menstruação por uso de contraceptivo)?				
	• Sim	0,96	1	1
	• Não			
	• Não tenho certeza			
	• Depende da situação			
Você acha que não consumir energia suficiente pode resultar na ausência de menstruação?				
	• Sim	1	1	1
	• Não			
	• Não tenho certeza			
Você acha que fraturas (fissuras ou pequenas quebras) ocorrem mais frequentemente em meninas/mulheres que não menstruam por 3 ou mais meses do que naquelas que menstruam regularmente? (excluindo gravidez ou ausência de menstruação por uso de contraceptivo)				
	• Sim	0,92	1	1
	• Não			
	• Não tenho certeza			
Você acha que a irregularidade ou ausência de menstruação está associada ao desenvolvimento de ossos mais fracos?				
	• Sim	0,96	1	1
	• Não			
	• Não tenho certeza			
Quais das opções a seguir podem aumentar o risco de um atleta contrair infecções, tais como gripe e resfriado? (por favor, marque todas as que se aplicam)				
	• Treino intenso com descanso inadequado	1	1	1
	• Consumo insuficiente de líquidos	0,88	1	0,95
	• Consumo insuficiente de energia	1	1	1
	• Não sei	1	1	1
Em sua opinião, quais das seguintes opções poderiam ser resultado da insuficiência crônica de energia? (por favor, marque todas as que se aplicam)				
	• Aumento do desempenho na corrida de velocidade	0,88	1	0,9
	• Diminuição do desempenho na corrida de velocidade	0,88	1	0,9
	• Mudanças no peso	1	1	1
	• Mudanças na composição corporal	1	1	1
	• Diminuição da força muscular	1	1	1
	• Aumento da força muscular	1	1	1
	• Diminuição da massa muscular	1	1	1
	• Aumento de lesões	1	1	1

 The pretest version (V2) of the questionnaire was applied to 12 female runners (mean age: 37.7 ± 11.3 years) who frequently participated in running events, eight running coaches (mean age: 38.8 ± 6.9 years), and 10 registered dietitians (mean age: 37.4 ± 11.4 years). Their feedback confirmed that the items were clear and understandable to 93% of respondents and adequate according to 96% of the sample. Given these findings, no modifications were necessary, and the pretest version (V2), presented in **Table 1**, was defined as the Brazilian version of the questionnaire to assess REDs knowledge (QC-REDs; see **Appendix 1**) to be tested using psychometric measures to accumulate validity evidence. 

## DISCUSSION

 The Brazilian version of the questionnaire to assess REDs knowledge demonstrated acceptable evidence of content and face validity, standing as the first tool in Brazilian Portuguese designed to assess knowledge about the signs, symptoms, and consequences of REDs. The main strength of this study lies in the testing of a needed, easy-to-apply, and easy-to-interpret instrument to measure knowledge about REDs. 

 The process of translation and cultural adaptation of the questionnaire to assess REDs knowledge was conducted carefully, following methodological recommendations widely recognized in the health field.^15 ,16^ The objective was to ensure that the Brazilian version maintained the quality and content of the original version proposed by Pai et al.^14^ Recommendations for conducting translation and cultural adaptation reflect best practices and raise expectations that the translated and culturally adapted instrument will be very similar to the original.^25^

 Based on the judgment of the expert committee, an excellent CVC of 0.97 was identified for the studied scale, with all items scoring above 0.8, supporting the retention of the questionnaire structure without modifications. Similarly, the original version of the questionnaire achieved a CVC greater than 0.8 based on expert evaluation.^14^ Content validity of a scale refers to whether the items are adequate to measure the construct it purports to measure in a relevant and representative way.^26^ The feedback provided by the evaluators is crucial for refining items before the scale is applied to larger samples.^26^

 According to the International Test Commission,^27^ an expert committee should be composed of individuals with different qualifications to ensure that all relevant aspects are addressed, including those that may be overlooked by others. In this context, a translator without expertise in health and sports participated in the committee. While this committee member focused solely on evaluating the linguistic clarity of the items, the practical pertinence and theoretical relevance were assessed exclusively by experts with knowledge of the construct, thus ensuring methodological rigor in the evaluation process. 

 Throughout the process, the original format of the questionnaire was preserved without removing or rearranging items. The adaptations focused on linguistic adjustments to enhance comprehension by the Brazilian population without affecting the real meaning of the expressions. The Brazilian version was developed following a content validity assessment by a committee of experts and was later validated by the target population. Given that content validity is fundamental to establishing other forms of validity, it should be prioritized in the instrument development process.^28^ Validity does not refer to the instrument itself but rather to the scores it produces when applied to a specific group of respondents for a particular purpose.^28^

 The fact that the pretest version underwent no modifications compared with the original version reinforces its fidelity to the original instrument. The involvement of the expert committee in the adaptation process is crucial for standardizing the items, ensuring clarity and ease of understanding, and maintaining the semantic equivalence of terms in relation to the original version.^29^

 The language and structure of the pretest version of the questionnaire were also validated by the target population (running coaches, dietitians, and female runners), who found the instrument to be clear, comprehensible, and adequate for their context. The absence of necessary modifications suggests that the adaptation was effective and well received by the participants. The importance of a test having high face validity is linked to the quality of the data collected and the participants’ experience.^21^ A questionnaire with high face validity naturally enhances data quality.^21^

 According to Magasi et al.,^30^ the perspective of the target population is important because they have direct, lived experience with the concept. In contrast, experts offer a broader view, having observed it across diverse individuals and contexts.^30^ Therefore, strong validity evidence requires balancing both perspectives (content and face validity) rather than favoring one over the other.^30^

 One limitation of this study is the lack of exploration of responses from health professionals and athletes beyond those included in the sample. While the participation of dietitians, running coaches, and female runners provided valuable insights into the clarity and adequacy of the QC-REDs, expanding the sample to include a broader range of professionals, such as physicians, physiotherapists, psychologists, and athletes from various disciplines, could offer a more comprehensive evaluation of the instrument’s applicability across different contexts. Additionally, future studies should investigate how the adapted version of the QC-REDs performs in diverse populations to ensure its validity across various sporting backgrounds. 

## CONCLUSION

 The findings of this study indicate that the Brazilian version of the QC-REDs demonstrated strong content and face validity. 

 This study contributes to the field by providing a rigorously adapted version of the QC-REDs for use in Brazil. The strong content validity and positive feedback from the target population support the questionnaire’s applicability and usability. In addition, this instrument fills a significant gap in the field by providing a valuable tool for researchers and professionals working with athletes and individuals at risk of REDs. 

 Future research focusing on psychometric validation to establish the robustness of this instrument in assessing REDs knowledge within the Brazilian context is in progress and will be reported soon. 

## Data Availability

Data supporting the findings of this study are available from the corresponding author upon reasonable request. Uyara Pereira de Maria.
