# Supplementary material for: Translation, cultural adaptation, and evidence of content and face validity of a questionnaire to assess REDs knowledge in Brazil: a cross-cultural validation study
Source: Sao Paulo Med J. 2026 Jul 17;144(3):e20253135. doi: 10.1590/1516-3180.2025.3135.08042026 (PMC13379200; doi:10.1590/1516-3180.2025.3135.08042026)
Supplement: Appendix 1 [file 1806-9460-spmj-144-03-e20253135-md1.docx]

Translation, cultural adaptation, and evidence of content and face validity of a questionnaire to assess REDs knowledge in Brazil: a cross-cultural validation study

Uyara Pereira de Maria^I^, Claudia Ridel Juzwiak^II^

Universidade Federal de São Paulo (Unifesp), Santos (SP), Brazil

^I^MSc. Doctoral Student, Interdisciplinary Postgraduate Program in Health Sciences, Universidade Federal de São Paulo (Unifesp), Santos (SP), Brazil.


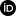
 <https://orcid.org/0000-0002-6903-073X>

^II^PhD. Professor, Department of Human Movement Sciences, Universidade Federal de São Paulo (Unifesp), Santos (SP), Brazil.


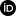
 <https://orcid.org/0000-0003-1101-0063>

**Appendix 1.** Brazilian version of the questionnaire to assess REDs knowledge [Questionário de Conhecimento da REDs (QC-REDs)]

| Você já ouviu falar de Baixa Disponibilidade de Energia?   - Sim - Não |
| --- |
| Você já ouviu falar de Deficiência Relativa de Energia nos Esportes?   - Sim - Não |
| Você já ouviu falar de Tríade da Mulher Atleta?   - Sim - Não |
| Ter um ciclo menstrual irregular costuma ser um sinal de que mulheres atletas/fisicamente ativas estão no auge da forma competitiva   - Verdadeiro - Falso - Não tenho certeza |
| Você acha normal mulheres atletas/ fisicamente ativas não menstruarem? (excluindo gravidez ou ausência de menstruação por uso de contraceptivo)?   - Sim - Não - Não tenho certeza - Depende da situação |
| Você acha que não consumir energia suficiente pode resultar na ausência de menstruação?   - Sim - Não - Não tenho certeza |
| Você acha que fraturas (fissuras ou pequenas quebras) ocorrem mais frequentemente em meninas/mulheres que não menstruam por 3 ou mais meses do que naquelas que menstruam regularmente? (excluindo gravidez ou ausência de menstruação por uso de contraceptivo)   - Sim - Não - Não tenho certeza |
| Você acha que a irregularidade ou ausência de menstruação está associada ao desenvolvimento de ossos mais fracos?   - Sim - Não - Não tenho certeza |
| Quais das opções a seguir podem aumentar o risco de um atleta contrair infecções, tais como gripe e resfriado? (por favor, marque todas as que se aplicam)   - Treino intenso com descanso inadequado |
| - Consumo insuficiente de líquidos |
| - Consumo insuficiente de energia |
| - Não sei |
| Em sua opinião, quais das seguintes opções poderiam ser resultado da insuficiência crônica de energia? (por favor, marque todas as que se aplicam)   - Aumento do desempenho na corrida de velocidade |
| - Diminuição do desempenho na corrida de velocidade |
| - Mudanças no peso |
| - Mudanças na composição corporal |
| - Diminuição da força muscular |
| - Aumento da força muscular |
| - Diminuição da massa muscular |
| - Aumento de lesões |
